# Integrated Analysis of Metabolites and Microorganisms Reveals the Anthracnose Resistance Benefits from Cyanidin Mediated by Proteobacteria in Tea Plants

**DOI:** 10.3390/ijms252111483

**Published:** 2024-10-25

**Authors:** Dandan You, Meiya Liu, Jianyun Ruan, Zhenhong Wang, Qunfeng Zhang

**Affiliations:** 1 Tea Research Institute, Chinese Academy of Agricultural Sciences, Hangzhou 310008, China; lay133i@163.com (D.Y.); liumeiya@tricaas.com (M.L.); jruan@tricaas.com (J.R.); 2 College of Resources and Environment, Xizang Agricultural and Animal Husbandry University, Linzhi 860000, China

**Keywords:** anthocyanin, anthocyanin metabolism, microbial diversity

## Abstract

Anthocyanins, key quality components of tea, act as an important bridge between plants and the environment due to their function on protecting plants from biotic and abiotic irritants. This study aimed to assess the interactions between anthocyanins metabolism and the environment. Purple (P) and green (G) leaves with different anthocyanin contents were inoculated with tea plant anthracnose. High-throughput metabolomics and 16S microbial diversity sequencing methods were used to screen the anthocyanin fractions of tea plant leaves responsive to anthracnose. The interconnections between metabolites and the resistance of phyllosphere microorganisms to fungal pathogens were then analyzed. The results showed that leaves with high anthocyanin content (0.14% of diseased area ratio) were less impacted by anthracnose infestation than leaves with low anthocyanin (3.12%). The cyanidin content decreased after infection in purple leaves (PR) and increased in green leaves (GR). The relative abundance of Cyanobacteria was suppressed by the significant enrichment of Proteobacteria after anthracnose infection in green leaves. However, there were no significant differences between these two groups of microorganisms in purple leaves. Collinear network analysis revealed a strong correlation between Cyanobacteria and Dihydrosorbinol and between Proteobacteria and cyanidin metabolites. Among them, OTU456 (Bosea) was identified as the key taxonomic group of bacterial communities in the green-infected leaf network. In summary, the anthracnose resistance benefits from cyanidin mediated by proteobacteria in tea plants. These results deepen our understanding of the regulation of secondary metabolism in tea plants and the formation of plant resistance.

## 1. Introduction

Tea is one of the major economic crops in China. The country is the global leader in terms of tea planting area and annual tea production [1]. With the rapid development of the tea industry, tea has become an important economic crop driving the economy [2]. Tea culture continues to penetrate Chinese society, and an increasing amount of the population chooses to consume tea. Thus, the production and promotion of tea consumption have been the focus of much attention, with tea selected among the six health beverages recommended by the World Health Organization. Tea has a variety of physiological benefits; in addition to antimicrobial, antioxidant, and anti-inflammatory properties, it also has the function of removing fat and lowering blood glucose, blood pressure, and blood lipids [3,4,5,6].

Tea plants grow mainly in subtropical and tropical regions [7]. The ecological environment in such warm and humid climates facilitates the growth and spread of various pathogens [8], resulting in a large number of tea plant diseases [9]. Anthracnose, a disease caused by *Colletotrichum*, is a pathogenic fungus that infects the young leaves, new shoots, flowers, and fruits of tea plants [10]. Small brown spots appear on the surface of leaves in the early stage of the disease, and dead spots are formed in the later stage. This reduces the yield and quality of tea [11] and leads to the death of the tea plants. As the cultivation area of winter tea gardens increases, the improper management and abuse of pesticides will inevitably lead to the deterioration of the ecological environment, prompting a large outbreak of tea anthracnose and causing economic losses. The widespread use of fungicides can produce serious side effects, such as drug resistance in pathogen populations [12], and is detrimental to human health and the environment.

Tea plants are rich in a variety of secondary metabolites with direct and indirect antimicrobial activities [13]. Moreover, anthocyanins are important secondary metabolites in tea plants [14]. Plants defend against pathogenic bacteria by regulating metabolic responses in vivo. Primary metabolites are mainly used as signaling substances to induce resistance and exist as precursors of secondary metabolites in plant disease resistance responses [15]. Secondary metabolites not only act as signaling substances to induce downstream antimicrobial production during plant defense but are also directly involved in the inhibition of pathogenic bacteria [16]. Numerous studies have reported that flavonoids have a broad antimicrobial spectrum against various pathogens [17]. Although some progress has been made in identifying the mechanisms by which plants develop resistance to anthracnose, information on the interactions between anthracnose and tea plants is limited.

Plants constantly interact with a variety of microorganisms in their natural environment [18]. While some microorganisms harm plants and trigger their defense responses, others are beneficial to plant performance. Numerous studies have demonstrated that microbiome assembly can be regulated by plant traits such as plant phenotypes, immune responses, and metabolic profiles [19]. Specific plant metabolites have beneficial or antagonistic effects on different microorganisms [20]. In this study, we analyzed anthocyanin metabolites in tea plant leaves in the context of the inter-foliar microbial community to explore the interactions between metabolites and microbial community responses to foliar pathogens. In particular, we screened potential anthocyanin compound fractions associated with microorganisms (especially pathogenic microorganisms), corroborated key anthocyanin fractions responding to tea plant resistance, and investigated the hidden interconnections between metabolites and interleaf microbial resistance to fungal pathogens.

## 2. Results

### 2.1. Analysis of Anthracnose Resistance of Tea Plant Leaves

**Figure 1:**
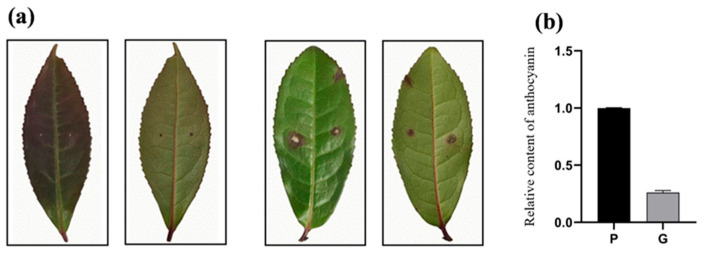
Leaf disease (**a**) and relative anthocyanin content (**b**) in green and purple leaves at 4 days of inoculation.

Four days after inoculation with the anthracnose strain, the leaves of healthy tea plants showed clear demarcation and disease symptoms on the epidermal surface, including yellow-brown irregular spots and lesion development (Figure 1a). These symptoms are consistent with those of anthracnose described above. The relative anthocyanin content (Figure 1b) in purple leaves (P) is higher than that in green leaves (G). Moreover, the diseased area (Table 1) of purple leaves (PR) after disease development was lower than that of green leaves (GR). The results also indicate that anthocyanin content is positively correlated with the resistance of tea plants to anthracnose pathogen infection. That means leaves with high anthocyanin content were less impacted by anthracnose infestation than leaves with low anthocyanin.

### 2.2. Screening and Characterization of Major Anthocyanin Fractions Responding to Anthracnose Resistance in Tea Plants

**Figure 2:**
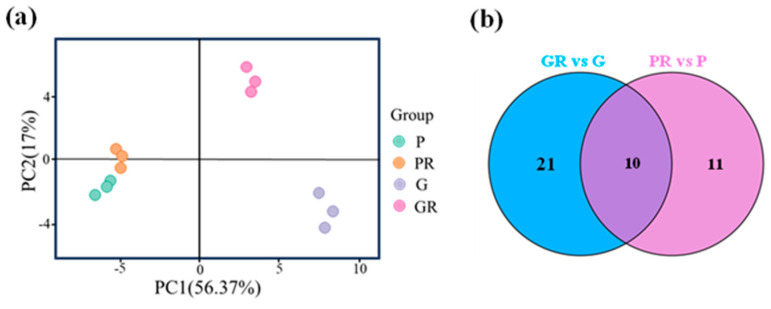
Analysis of anthocyanin metabolism in different leaves; (**a**) principal component analysis; (**b**) Venn plot.

Anthocyanin metabolome assay was performed on four groups of tea plant leaf samples, and a total of 64 anthocyanin-related metabolites were detected. These metabolites were subjected to principal component analysis (PCA). The two PCA axes (Figure 2a) contributed 56.37% and 17% to the variance of the data, respectively. PR, G, and GR all showed significant separation on PC1 and PC2, while P and PR did not exhibit any separation on these two axes. This indicates that there was a significant difference between the metabolites of PR and GR tea plant leaves. The PCA results for anthocyanin show that P and PR metabolites did not differ significantly, and the response of purple leaves to the pathogen infestation after inoculation was minimal. G and GR metabolites differed significantly, suggesting that the green-leaf metabolites responded to the pathogen after inoculation.

**Figure 3:**
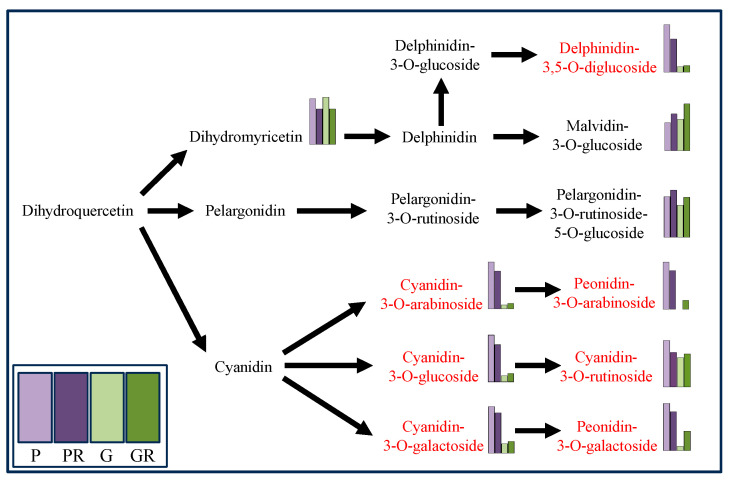
Differential anthocyanin metabolic pathway (Red marks indicates metabolites decreased in purple leaves but increased in green leaves after infection).

Metabolomics analysis was employed for the targeted determination of anthocyanin metabolites in P and G and the leaves of PR and GR after infection with anthracnose. Using VIP > 1.0 and *p*-value < 0.05 as the screening criteria, a total of 10 significantly different metabolites were screened (Figure 2b). Among these, the contents of seven key flavonoid metabolites (cyanidin-3-O-glucoside, cyanidin-3-O-arabinoside, cyanidin-3-O-galactoside, cyanidin-3-O-rutinoside, Delphinidin-3,5-O-diglucoside, Peonidin-3-O-galactoside, and Peonidin-3-O-arabinoside) exhibited the same change trend after pathogen infestation, which manifested as a decrease in the PR content and an increase in the GR content (Figure 3).

### 2.3. Interleaf Microbial Community Diversity of Healthy and Diseased Tea Plant Leaves

By amplicon sequencing, where the chloroplast reads were removed, the V4–V5 regions of 16S rDNA sequences yielded original optimized sequences of 860,528 entries, with 322,251,004 bases and an average sequence length of 374 bp.

**Figure 4:**
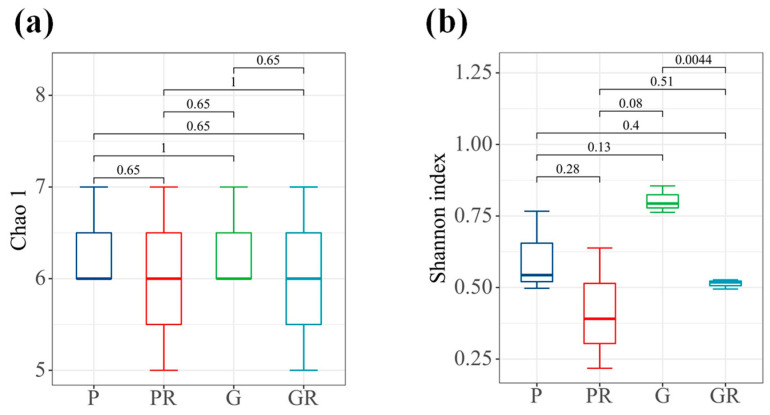
Interleaf diversity index map of tea plants. (**a**) Chao1 index. (**b**) Shannon index.

In terms of bacterial diversity (Figure 4b), there were significant differences between the samples of G and GR (*p* < 0.05). No significant differences were observed between the richness indices of the bacterial communities of PR and GR. Furthermore, the diversity indices of the bacterial communities decreased in PR and GR compared with P and G. Similarly, there were no significant differences between the richness indices of the bacterial communities of P and PR and the diversity indices of the bacterial communities of P and PR.

#### Interleaf Microbial Community Structure and Composition of Healthy and Diseased Leaves

**Figure 5:**
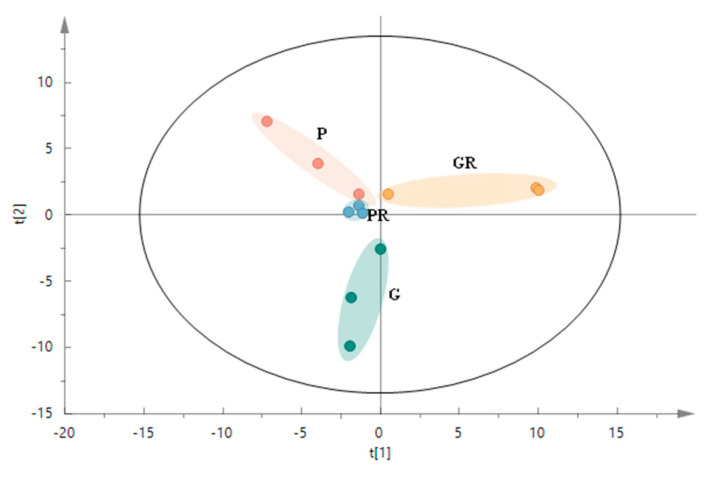
PCA score plot of microbial communities in the structure of the interleaf microbial communities after the leaf inoculation treatments.

The structure of microbial communities was analyzed and visualized using PCA (Figure 5) to reflect the changes in the structure of the interleaf microbial communities after the leaf inoculation treatments. The P and G treatments had a clear tendency to segregate on the second principal component (R2x[2] = 0.233), and the GR treatment generally segregated from the P, PR, and G treatments on the first principal component (R2x[1] = 0.299). PCA analysis revealed that the PR and P groups were closer to each other. This indicates that in terms of species composition structure, the interleaf microbial community structures of the PR and P groups were similar, while that of the G group differed from those of the GR and P groups. This is due to the greater impact of the *Colletotrichum* infestation on the structure of the interleaf microbial community.

**Figure 6:**
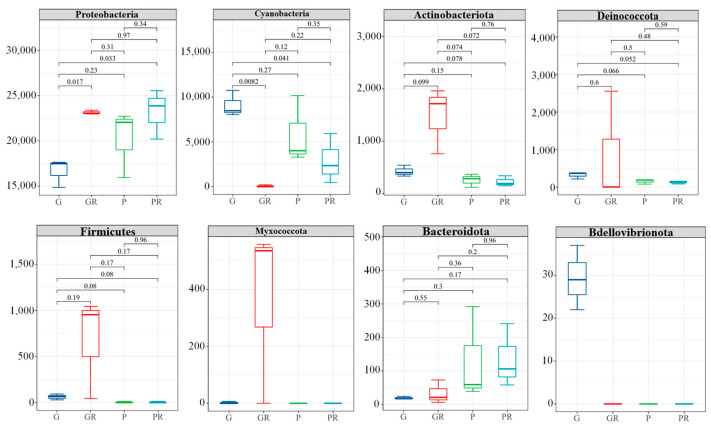
Structural composition of the horizontal community of the interleaf bacterial phylum.

After the inoculation of pathogenic bacteria, 16S rDNA sequencing of bacteria infected by leaves showed differences in the phyllosphere microbial community composition at the phylum (Phyla) level (Figure 6). There were no significant differences in PR and GR in low-abundance taxa (e.g., Decobacteria, Firmicutes, and Bacteroidota). However, in G and GR, Proteobacteria was significantly enriched in GR (*p* < 0.05), while the opposite was observed for Cyanobacteria. The relative abundance of Cyanobacteria increased significantly in G and decreased significantly in GR. In P and PR, the relative abundance of Cyanobacteria in PR was also reduced. This may be attributed to the infection of bacteria in the leaves and the competition of nutrients between bacteria, which reduced the Cyanobacteria content. This shows that following the infection of anthracnose disease, anthracnose occupies the living space of other fungi.

### 2.4. Response of the Co-Occurrence Network to Leaf Infestation at Two Anthocyanin Content Levels

**Figure 7:**
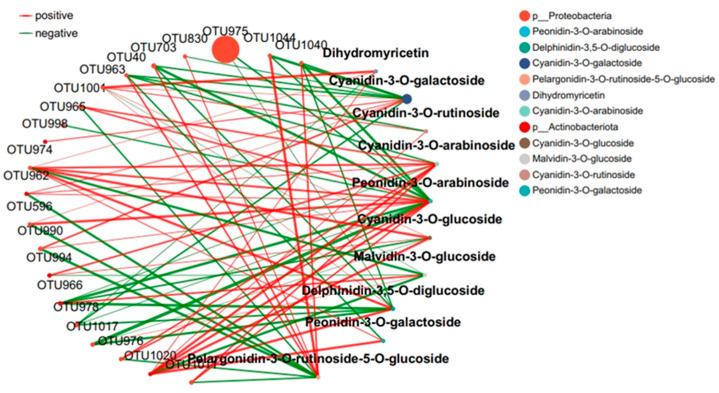
Collinear network of anthocyanin and microbial communities in purple tea leaves.

**Figure 8:**
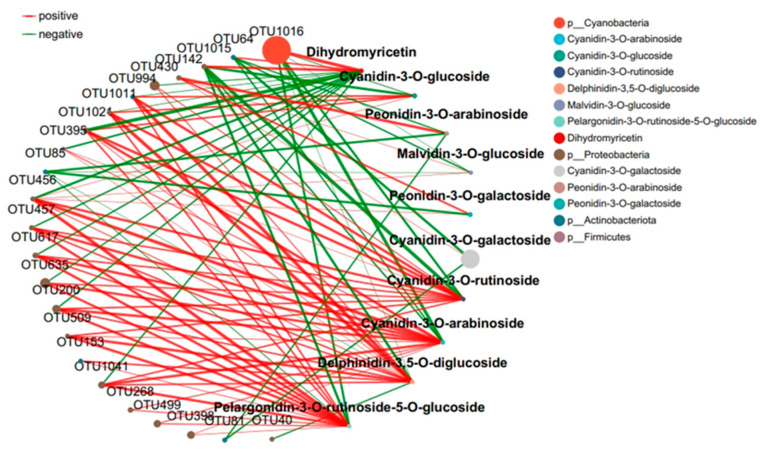
Collinear network of anthocyanin and microbial communities in green tea leaves.

The interaction between microbial communities was analyzed based on the OUT co-occurrence network (Figure 7 and Figure 8). GR had a more complex and stable collinear mode than PR. This is reflected in the higher topological parameters of the OTU (especially the increased number of nodes and links in the network). Compared with simple network relationships, complex networks have a higher potential for resource and information transmission. They also tend to have a more stable microbial community. Here, due to the increased content of cyanidin metabolites in GR (FIG. 8), there was a close relationship between Proteobacteria and cyanidin metabolites, and higher network complexity and microbial growth were observed under GR. Microbial interactions are closely related to anthocyanin metabolites.

The interaction between microbial communities and metabolites was mainly positive (>60%). This emphasizes the importance of the synergy between microbial communities and metabolites in the resistance of tea plants to pathogen infection. Positive interactions between microbial communities and metabolites indicate cooperative relationships or niche overlaps, while negative interactions indicate competitive relationships or niche separation. In this study, the OUT with the highest centrality value was regarded as the key taxonomic group. OTU456 (Bosea) and OTU974 (Sphingomonadaceae) were identified as the key taxa of bacterial communities in the leaf network of GR and PR, respectively.

## 3. Discussion

Plants produce various chemical stimuli to recruit beneficial microorganisms or alter their microbial communities in response to pathogen infection [21,22]. This phenomenon is denoted the “call for help” strategy, and plants actively cooperate with microorganisms to deal with diseases. During the attack of pathogens on plant leaves, the disease-inhibiting microbiome gradually accumulates in the phyllosphere [23]. This study found that anthocyanin components play an important role in the severity of tea anthracnose. The flavonoid synthesis pathway in plants is the most studied secondary metabolic pathway in current research. Flavonoids have important physiological significance in plants and play a key role in plant resistance to pests and diseases and signal transmission with microorganisms, as well as numerous other factors [24,25]. The flavonoid biosynthesis pathway is directly related to plant stress resistance. In this study, leaves with high anthocyanin content exhibited the lowest diseased area ratio after infection by *Colletotrichum* gloeosporioides. When green leaves were infected by pathogens, the cyanidins were synthesized in large quantities to resist pathogens. Recent studies have shown that primary and secondary metabolites can regulate the phyllosphere microbiota [26]. These compounds are important nutrient sources for phyllosphere microorganisms and can affect the development stage-specific core microbiota, thereby regulating the phyllosphere microbiota. For example, metabolites such as flavonoids, lignin precursors, quaternary ammonium salts, terpenoids, etc. are enriched with the expansion of lesions, have a positive regulatory effect on Rhynchogastremataceae and Actinomycetales, and play an important role in the aggregation of the phyllosphere microbial community [26]. This suggests that plants can recruit beneficial microorganisms by releasing specific compounds.

Flavonoids are the major secondary metabolites of tea plants and substantially impact tea quality. Flavonoids act as an important bridge between plants and the environment, protecting plants from biotic and abiotic irritants [13,17]. This study investigated the relationship between anthocyanin components and the phyllosphere microbial community on the inhibition of tea anthracnose. Numerous studies have reported that flavonoids have a broad antimicrobial spectrum against various pathogens [17]. Anthracnose was observed to be closely related to the diversity, community composition, and inferred anthocyanin–microbial interaction of the phyllosphere microbial community. Alpha diversity index analysis showed that the bacterial community diversity index of green (G) and green infected (GR) leaves was significantly lower than that of green infected (GR) leaves. Compared with the bacterial community diversity index of purple (P) and purple infected (PR) leaves, there was no significant difference in the diversity index of purple infected (PR) leaves, indicating that purple (P) leaves had strong resistance. Anthracnose can affect the synthesis of flavonoids in tea plants [27]. Several studies have shown an increase in the levels of anthocyanins and related compounds in plants under external environmental stimuli [14,15] Our results verify the previous observation that the diversity of the phyllosphere microbial community was reduced due to the infection of the anthracnose pathogen.

In their long-term evolution, plants have developed complex interrelationships with microorganisms that play an important role in plant growth and health [18]. In this study, Proteobacteria, Cyanobacteria, and Actinobacteria were identified as the dominant bacteria in the phyllosphere of healthy and susceptible tissues of tea plants after the occurrence of anthracnose. Although the sampling time of the two bacteria was different, after inoculation with pathogens, Proteobacteria in green leaves was significantly enriched (*p* < 0.05), while the relative abundance of Cyanobacteria was inhibited. However, there were no significant differences between the two microorganisms in purple leaves. While some microorganisms harm plants and trigger their defense responses, others are beneficial to plant performance [19]. These taxa are generally considered to be beneficial microorganisms for plants [28]. Proteobacteria are a group of protists that are widely present in the natural environment. They have the ability to engulf bacteria and other organic matter and can adapt to different environments and lifestyles through morphological changes. They are both important microbial groups in nature [21,22]. Moreover, Proteobacteria can produce medicinal substances with antibacterial properties and resistance to disease and swelling pain. Actinomycetes are well-known antagonistic microorganisms that fight against numerous pathogens as they can produce a variety of antibiotics, secrete cell-wall-degrading enzymes, and/or induce host (plant) resistance [28].

Plants constantly interact with a variety of microorganisms in their natural environment [18]. In this study, the leaves of the green inoculation treatment exhibited more complex and stable collinear patterns than the leaves of the purple inoculation treatment. In the co-occurrence network constructed by the OTUs, a higher network complexity and microbial growth were observed under the green-leaf inoculation. A strong correlation was observed between Cyanobacteria and Dihydrokaempferol in the collinear network, and between Proteobacteria and cyanidin metabolites. This can be attributed to the fact that tea plants are rich in a variety of secondary metabolites with direct and indirect antimicrobial activities [13]. OTU456 (Bosea) was identified as the key taxonomic group of bacterial communities in the green-infected leaf network. The genus Bosea is a Gram-negative bacterium belonging to the Alphaproteobacteria Bradyrhizobiaceae [29]. The strain of B. bostrychophila is reported to have good inhibitory effects on 11 pathogenic fungi (*Colletotrichum* gloeosporioides, Botrytis cinerea, Phomopsis microsporioides, Rhizoctonia cerealis, Rhizoctonia solani, Alternaria solani, Lily root rot, Fusarium verticillioides, Verticillium dahliae, Fusarium oxysporum f.sp.niveum, and Alternaria solani) [29]. Thus, it is concluded that Boschella has an inhibitory effect on anthracnose in green leaves.

## 4. Materials and Methods

### 4.1. Pathogen Identify and Inoculation of Colletotrichum gloeosporioides

Healthy fully expanded purple (P) and green (G) leaves of tea plants (*Camellia sinensis* (L.) O. Kuntze) with different anthocyanin contents were selected as the test materials. The test strain was extracted from the tea garden of the Tea Research Institute of the Chinese Academy of Agricultural Sciences, Hangzhou, China, as typical diseased tea plant anthracnose. The disease spots were semi-circular or irregularly shaped, with obvious demarcation, producing watery yellow-brown dots, and the expansion of the spots changed from brown to scorched yellow and finally grayish-white. The pathogen was identified at the species level after isolation and purification from the diseased tea leaves by the tissue isolation method. Genomic DNA was extracted and amplified from the purified pathogen, and its internal transcribed spacer region (ITS) was sequenced using primers ITS1 and ITS4. The pathogen ITS sequence showed 99% sequence similarity to that of *Colletotrichum*. Therefore, the fungus was identified as *Colletotrichum* based on its morphological and molecular characteristics.

Purple (P) and green (G) leaves (three biological replicates) with different anthocyanin contents were selected for inoculation with the identified *Colletotrichum* gloeosporioides. Samples were taken 4 days after inoculation for testing. In the inoculation test of anthracnose, the infection time of leaves of different colors and the diseased area ratio of leaves at the end of the test were used as indicators of the resistance of tea plant varieties with different anthocyanin contents to tea plant anthracnose. The diseased area ratio was calculated by the pixel counting method. Photoshop CS5 (Adobe, San Jose, CA, USA) was employed to select the total and diseased leaves, and the number of pixels of each was taken as the total and diseased area, respectively. The disease area ratio (example 1) was calculated using the following formula:

(1) $$\mathrm{Disease}\;\mathrm{area}\;\mathrm{ratio}=\;(\frac{\sum_{i=1}^{6}\mathrm{Diseased}\;\mathrm{area}}{\sum_{i=1}^{6}\mathrm{Total}\;\mathrm{area}})\%\;$$

### 4.2. Determination of Anthocyanins and Metabolites in Tea Leaf by Ultra-Performance Liquid Chromatography (UPLC) Coupled to Mass Spectrometry

The biological samples were vacuum freeze-dried and ground (30 Hz, 1.5 min) to powder form using a ball mill; 50 mg of the powder was extracted with 500 μL of 50% aqueous methanol containing 0.1% hydrochloric acid by following the method proposed by Zhang et al. [30]. Extracts were then analyzed with UPLC-MS/MS (ExionLC™ AD, Foster City, CA, USA; QTRAP^®^ 6500+, Foster City, CA, USA) as described in Zhang et al. [31].

### 4.3. Endosphere Microecology with 16S Microbial Diversity Sequencing of Endophytic Mycobiota

The leaf surface was sterilized by washing the samples with sterile water for 30 s following the method described previously [32]. Microbial genomic DNA was directly extracted by a DNA extraction kit (Beijing Huatuyang Biotechnology Co., Ltd., Beijing, China) and detected by NanoDrop2000 as described previously [33,34]. An Illumina library was constructed according to the method described by Yang et al. [35].

## 5. Conclusions

In summary, this study reported the changes in metabolites and endogenous microbial community information in tea leaves related to anthracnose. We found that the relative content of anthocyanin was positively correlated with anthracnose resistance, and the Proteobacteria in leaves was significantly correlated with anthocyanin, indicating that Proteobacteria exhibits disease resistance. The results proved that anthracnose resistance was associated with the variation of anthocyanin in tea plants, which may be related to the presence of Proteobacteria microorganisms. Our results revealed the interaction of tea tree anthracnose, anthocyanin metabolites, and the microbial community in tea plants, with the three elements influencing and balancing each other.

## Figures and Tables

**Table 1:** Diseased area ratio of green and purple leaves.

	Diseased Area (Pixels)	Total Area (Pixels)	Diseased Area Ratio (Diseased Area/Total Area)%
Green leaf	16,376	525,364	3.12
Purple leaf	570	413,994	0.14

## Data Availability

The data underlying this article will be shared on reasonable request to the corresponding author.
